# Autism Diagnostic Assessments for Gender-Diverse Individuals: A Modified Delphi Study of Clinician Experts in the Fields of Autism and Gender Diversity

**DOI:** 10.1016/j.jaacop.2025.10.001

**Published:** 2025-10-10

**Authors:** Kate Cooper, Anna I.R. van der Miesen, Meng-Chuan Lai

**Affiliations:** a University College London, United Kingdom; b Centre for Applied Autism Research, University of Bath, United Kingdom; c Amsterdam University Medical Centers, Location Vrije Universiteit, Center of Expertise on Gender Dysphoria, Amsterdam, the Netherlands; d Margaret and Wallace McCain Centre for Child, Youth & Family Mental Health and Azrieli Adult Neurodevelopmental Centre, Campbell Family Mental Health Research Institute, Centre for Addiction and Mental Health, Toronto, Ontario, Canada; e Temerty Faculty of Medicine, University of Toronto, Toronto, Ontario, Canada; f Faculty of Arts and Science, University of Toronto, Toronto, Ontario, Canada; g The Hospital for Sick Children, Toronto, Ontario, Canada; h Autism Research Centre, University of Cambridge, Cambridge, United Kingdom; i National Taiwan University Hospital and College of Medicine, Taipei, Taiwan

**Keywords:** autism, autism assessments, Delphi study, gender diversity, gender identity

## Abstract

**Objective:**

There is limited evidence-based guidance about how autism diagnostic assessments should be conducted for gender-diverse people. This study aimed to integrate expert knowledge on key clinical considerations for these assessments.

**Method:**

A modified Delphi study was conducted. World experts in the field (N = 21) were invited to complete 2 rounds of surveys. Survey one collected open-text responses about key clinical considerations when conducting diagnostic assessments, structured around the *DSM-5-TR* criteria for autism, across age ranges. Experts were asked to rate the importance of each consideration they listed. A content analysis was conducted to synthesize and collate similar considerations, alongside descriptive statistics of importance ratings. Survey two presented the resulting considerations and mean importance ratings, with experts rerating their importance. Statements rated as at least “important” and that had an SD of less than 1.0 were reported.

**Results:**

Round one resulted in 65 individual statements, of which 37 met our definition for reporting. These statements, summarizing expert opinions, were categorized as being general considerations for assessments, linked to the *DSM-5-TR* autism criteria (A-E), or practical considerations for working with the gender-diverse population. They highlighted areas to be considered during assessments, such as ways in which the features of autism may intersect with gender diversity, and practical considerations for increasing comfort and engagement of gender-diverse individuals undergoing an autism assessment.

**Conclusion:**

The summary of expert opinions provides preliminary considerations for clinicians working in this field and for researchers to use as hypotheses for empirical investigations.

## Plain language summary

Gender-diverse people are more likely to be diagnosed with autism or have autistic traits. In this study, the authors conducted surveys of 21 experts in conducting autism assessments in youth from around the world. The authors summarized expert opinion including general considerations for assessment, autism diagnostic criteria, and working with gender-diverse populations.

Gender-diverse people are more likely to be autistic and to have elevated autistic traits than cisgender people.^1^ In this article, we use the term gender diversity to refer to people who identify as transgender, nonbinary, gender nonconforming, or gender questioning. We use the term autism to refer to autism spectrum disorder, as defined in *DSM-5-TR*, to be respectful of the preference of the autistic community.^2^ The clinical care needs of this intersectional group can be heightened and complex.^3^^,^^4^ An interplay of biological, psychological, and social factors is hypothesized to contribute to this intersection.^3^ However, research about the intersection of autism and gender diversity is in its infancy, and we currently have a limited understanding of how to disentangle autism- and gender-related differences across development. Guidance focused on clinical care needs is scarce, especially regarding how an autism assessment should be optimized for gender-diverse individuals.^5^

Autism diagnostic assessments involve complex clinical decision making. Autism is characterized by differences in social communication and interaction and restricted, repetitive behaviors, interests, and activities (RRBIs).^6^ Critically, the diagnostic process includes differential diagnosis; according to the current diagnostic criteria for autism spectrum disorder, the features of autism should not be better accounted for by other conditions.^6^ Therefore, clinicians must assess for the presence of other mental health or developmental experiences that may account for the individual’s behavioral profile. This includes understanding social experiences and adverse life events, alongside developmental, physical, and mental health conditions that may have affected a person’s developmental trajectory. This is a highly complex process with multiple influences from sociocultural and contextual factors,^7^^,^^8^ as well as clinician views, knowledge, and training.^9^

There is some evidence that being gender diverse can influence key developmental experiences known to overlap with the features of autism and that therefore require particular attention during an autism diagnostic assessment, including adverse childhood experiences, social anxiety, and sensory responses.^10^, ^11^, ^12^ For example, compared with other children, gender-diverse children are more likely to experience negative social experiences such as being bullied.^10^ Qualitative research has also shed light on how being gender diverse^13^ and having gender dysphoria^14^ are experienced in autistic youth, demonstrating overlapping phenomenology in development. A desire to be perceived as one’s gender by others can lead to hypervigilance during social interactions, focused on reducing the likelihood of being misgendered, which can contribute to behavioral and cognitive processes related to social anxiety.^11^ A recent meta-synthesis^15^ found that some autistic and gender-diverse individuals may spend time masking to appear both neurotypical and cisgender. There was a sense of self-blame for not meeting societal standards in terms of typical gender expression. There has also been debate as to whether autistic traits may change in individuals accessing transgender health services. Two of 3 available longitudinal studies indicated that autistic traits remain stable 12 months after starting gender-affirming hormone treatment in adults (n = 118)^16^ and puberty blockers in adolescents (n = 95),^17^ and 1 study found a significant reduction in autistic traits after 12 months of gender-affirming hormone treatment in adults (n = 62).^18^

There are barriers to gender-diverse individuals accessing autism assessment and support.^19^ Autism may be diagnosed later in gender-diverse people compared with cisgender people,^20^^,^^21^ similar to the inequities faced by many individuals assigned female at birth compared with individuals assigned male.^22^ This is likely due in part to the historical focus on and understanding of autism as it manifests in individuals assigned male at birth, boys and men, and associated gendered expectations and stereotypes.^21^ There is increasing knowledge on how to recognize the nuanced phenotypes of autism for individuals across binary sexes and genders, especially in individuals assigned female, girls and women.^23^ However, further research and clinical exchanges are required to build consensus around whether and how knowledge can be practically applied to gender-diverse individuals more broadly, especially considering the substantial service needs in individuals seeking autism assessments who identify as gender diverse.^16^

Integrating current clinical expertise is an important step in developing the evidence base for future empirical work. Although initial clinical guidelines exist for supporting autistic gender-diverse individuals,^24^ no research has yet looked at how best to conduct autism diagnostic assessments for gender-diverse individuals. Gender and autism services globally have gained diverse clinical insights from their experiences in the assessment of autism in gender-diverse individuals across different ages, but, to our knowledge, no key considerations on autism assessment in gender-diverse individuals have been systematically summarized yet.

To address this gap, the current study aimed to integrate expert knowledge and summarize preliminary key considerations for diagnosing autism in gender-diverse individuals. We aimed to produce consensus statements to guide clinical decision making during autism diagnostic assessments for gender-diverse individuals and for researchers to use as hypotheses for empirical investigations.

## Method

### Methodological Approach

We used the Delphi procedure, a standardized and well-studied multistep survey method for deriving consensus among clinician experts.^25^^,^^26^ It ensures confidentiality and allows each expert to give their input without being influenced by others. By using an iterative process, it helps prevent issues that can arise in live group discussions, such as defending one’s ideas or relying too much on the input of senior members. This method fosters unbiased and well-rounded consensus among the experts involved. The authors were independent from the Delphi expert panel for the proposed study.

### Expert Panel

A panel of international expert practitioners was recruited (N = 21) (Table 1). One participant did not complete the first survey, and a different participant did not complete the second survey (ie, 20 participants completed each survey). All experts were experienced in autism diagnostic assessments as defined by the following inclusion criteria: qualified autism diagnostician for children and youth and/or adults, with appropriate qualifications in a core health profession including training in developmental conditions/disorders and administration of standardized autism assessments, and a minimum of 3 years of experience conducting autism diagnostic assessments with gender-diverse people.

**Table 1:** Demographics of Experts

Characteristic	n	(%)
Gender identity		
Man	8	(38)
Woman	13	(62)
Nonbinary	0	(0)
Other gender identity	0	(0)
Gender diverse/transgender		
Yes	2	(10)
No	19	(90)
Autistic/neurodivergent		
Yes	2	(10)
No	18	(86)
Prefer not to answer	1	(5)
Professional background		
Practitioner psychologist (clinical, counseling, or educational psychologist or neuropsychologist)	12	(58)
Physician	8	(38)
Not answered	1	(5)
Highest professional qualification		
Clinical or educational psychology doctorate	7	(34)
Medical doctorate	5	(24)
PhD	4	(19)
Postgraduate degree (eg MSc)	5	(24)
No. years conducting autism assessments with gender-diverse individuals		
3-5	6	(29)
6-10	7	(33)
11-15	4	(19)
>15	4	(19)
Estimated no. autism assessments with gender-diverse individuals		
<10	1	(5)
10-19	6	(29)
20-29	3	(14)
30-49	3	(14)
50-99	3	(14)
>100	5	(24)
Age group^a^		
Children	14	(67)
Adolescents	17	(81)
Adults	16	(76)
Continent of practice		
South America	1	(5)
North America	4	(19)
Asia	2	(10)
Europe	14	(67)

Note: ^a^Participants could endorse more than 1 age-group specialty.

Ten percent of participants were gender diverse, and 10% were neurodivergent. Among participants, 71% had 6 years or more of experience conducting autism assessments with gender-diverse individuals.

### Recruitment

Experts were contacted through personal contacts of the authors of this report, all of whom met the above-listed criteria. Experts were further identified by contacting key authors in the clinical research in this field and through snowballing.

### Procedure

**Figure 1:**
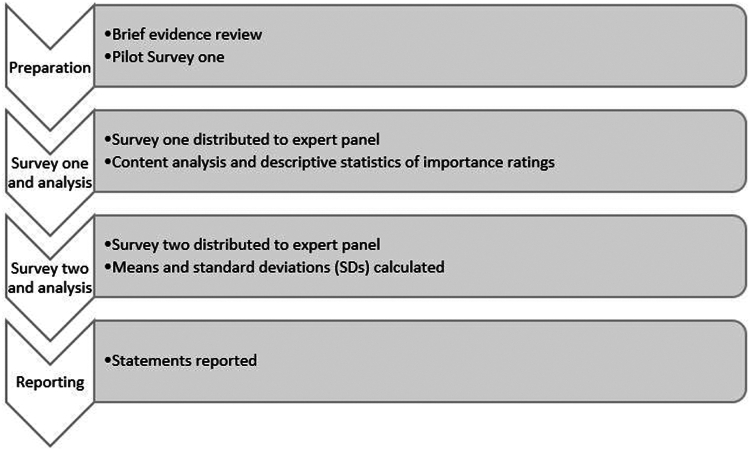
Procedure Overview

The study followed a modified 2-round Delphi procedure^21^ due to limited time and available resources and in acknowledgment of the clinical and research commitments of all experts who agreed to participate (Figure 1). To prepare, a brief evidence review was conducted, focused on 2 key systematic reviews.^1^^,^^15^ These reviews and authors’ knowledge were used to create the first survey. In survey one, experts were asked to list important considerations when conducting autism assessments with gender-diverse individuals and rate their importance. We then conducted a content analysis of expert responses to generate a list of statements for survey two. In survey two, experts were asked to rate each of the statements generated for their importance. Following this, means and SDs of each statement were calculated for the group. Finally, in the reporting phase, statements were categorized and reported based on whether they reached an a priori definition of consensus, as well as being rated as at least “important” by experts.

### Survey Development and Piloting

The systematic review findings were carefully considered and assimilated into the design of the Delphi surveys. Kallitsounaki and Williams^1^ provide support for the need for research to support high-quality autism assessments in gender-diverse individuals in clinical settings. The meta-synthesis by Moore *et al.*^15^ highlights the importance of allowing experts to make suggestions for capturing subjective experiences about the intersection of autism and gender diversity.

The survey instruments were written and piloted among the authors, who are practicing clinician-researchers in this field (2 physicians and 1 clinical psychologist, based in the Netherlands, Canada, and the United Kingdom), and discussed with a gender-diverse autistic adult to ensure that they mapped onto their lived experience. The survey items were structured according to the *DSM-5-TR* criteria for autism spectrum disorder with the understanding that this is the most commonly used framework internationally as the basis for autism diagnostic decisions.

### Survey One

The first survey was sent in October 2023, and data collection ended in February 2024. It consisted of open-ended questions with requests for relevant considerations for each part of the *DSM-5-TR* criteria (eg, A1, A2). For example, the prompt for the first part of the survey was “Please describe below how being gender-diverse can interact with the specific features of autism by history and current behavior, focusing on the *DSM-5-TR* Criteria A1. Please rate the importance of each point using a Likert scale below. By importance we mean how much weight it would carry within the diagnostic decision making.” Each aspect of the *DSM-5-TR* diagnostic criteria was summarized as a prompt for the experts completing the survey.

There were additional open-ended questions at the end of the survey:

1. “What are other key practical considerations in the individual’s journey through a service when undertaking an autism assessment with a gender-diverse individual?”
2. “What are other key considerations relating to assessment process and components when undertaking an autism assessment with a gender-diverse individual?”
3. “What are other key considerations relating to particular gender experiences when undertaking an autism assessment with a gender-diverse individual?”
4. “What are other key considerations relating to your specific health care context when undertaking an autism assessment with a gender-diverse individual?”
5. “Are there other key considerations not listed above you take into account when undertaking an autism assessment with a gender-diverse individual?”

Experts were asked to list separate considerations in response to each question, including open questions. They then were asked to rate the importance of the consideration they had written on a 7-point Likert scale, with the prompt “Rate the importance of each point using the Likert scale below. By importance we mean how much weight it would carry within the diagnostic decision making.” Response options ranged from “extremely unimportant” to “extremely important” (1-7).

A content analysis was conducted using NVivo software (Lumivero, Denver, CO). The first author (K.C.) gained familiarity with the expert statements by adding them to a single NVivo project and reading and rereading them closely. Statements that were judged as describing similar considerations were grouped together. Statements were judged as similar when they had semantic and conceptual coherence. In the first instance, this was when experts were expressing essentially the same idea using different words, for example, “practicing/imitating specific behaviors to express more in line with experienced gender” and “Transpersons try to mimic how their peers behave in different situations so that they will pass as boys or girls.” In the latter case, statements were grouped together when they were similar ideas but covered multiple areas of development or behavior. For example, “GV [gender variant] people have all kinds of difficulties because of discrimination and rejection in a [sic] several areas, that are not a result of an impairment in social skills” and “feeling uncomfortable in social interactions given experienced incongruence” were drawn together with other conceptually coherent expert comments to create the statement “Assess for whether social withdrawal is due to experiences of bullying and rejection and dysphoria (eg, about voice, body) or autism,” which encapsulates multiple ideas about causes of social withdrawal in this population. Statements were grouped together under codes in NVivo and were given a label that formed a succinct and clear summary statement. They were written as practice statements, for example, “Clinicians should assess if unusual quality of speech, eg, tone, rate, modulation, is due to discomfort with voice and/or autism.” The first author presented the content analysis findings to the other authors for verification, discussion, and modification. Disagreements on categorization of expert statements were resolved through discussion among the authors, using the majority rating where consensus could not be reached. Means and SDs of the importance ratings were calculated for each statement for presentation in survey two.

### Survey Two

The second survey was sent in April 2024, and data collection completed in August 2024. It included statements that were mentioned by more than 1 expert and that were rated on average at least “slightly important.” In an amendment from the original plan, some statements were included even if mentioned by a sole expert if they were consistent with more generalized statements listed by other experts but added clinically useful detail as agreed by the authors.

In this round, experts were shown the average importance ratings for each statement. They were then asked to rate the degree to which they agree with each statement. If they wished to add a clarification, they could add additional information in an open-text box.

### Reporting

Finally, means and SDs of importance ratings for each statement in survey two were calculated. The definition of consensus as provided by Franc *et al.*^27^ was used. This states that when using a 7-point Likert scale, if the SD is <1.0, then consensus has been reached. As only 2 rounds of surveys were planned, we had intended to report statements in the following categories: consensus reached (those meeting the criteria above), endorsed by the majority (statements endorsed as at least “important” by >50% of experts), and not endorsed (statements not meeting either criterion outlined in the first 2 categories). However, based on the feedback from experts, and because many statements had a wide range of importance ratings, we amended the plan to report the statements more conservatively and precisely. We therefore report in “Results” average rating (from “extremely unimportant” to “extremely important”), consensus reached (those meeting the criteria defined by Franc *et al.*^27^), and percentage rated “important” or higher.

## Results

Survey one was completed by 20 experts (95% completion rate), generating their own considerations for autism diagnostic assessments with gender-diverse individuals based on the *DSM-5-TR* criteria for autism spectrum disorder. Content analysis generated 65 separate statements (Table 2), which were grouped in the following categories: general considerations regarding autism assessments; across A criteria; A1; A2; A3; across B criteria; B1; B2; B3; B4; criterion C; criterion D; criterion E; practical considerations. The statements that were rated on average as “important” or “extremely important” (scored 6 or 7) and for which a consensus was reached (SD <1.0) are in boldface in Table 2.

**Table 2:** Considerations From Content Analysis and Average Survey Two Rating

Statement^a^	Min	Max	Mean	SD	Average rating^b^	Consensus reached?	Percentage rated important or higher (%)
					


General considerations							
**Particular attention must be paid to disentangling autism from (co-)occurring developmental (eg, intellectual disability, ADHD) and mental health needs. This may include considering the roles of depression, anxiety (especially social anxiety), OCD, personality disorders, trauma responses, and PTSD and how these influence history, current presentation, and functioning.**	5.00	7.00	6.30	0.57	Important	Yes	95
**Clinicians must maintain awareness of their own and societal gender stereotypes. They should also be aware of sex- and gender-related differences in social communication styles, social relationships, and restricted and repetitive behaviors and sensory sensitivities.**	5.00	7.00	6.10	0.55	Important	Yes	90
**Clinicians should aim to understand the extent to which masking is autism and/or gender related (ie, modifying one’s gender expression to conform to social norms regarding typical gender behavior—to be seen as gender conforming). Because gender masking can impact social development, clinicians should explore the impact of any gender-related masking on sense of self and social development.**	3.00	7.00	5.70	0.92	Important	Yes	75
**Clinicians should assess if rigid thinking about gender could be linked to gender diversity and gender-related needs or is more related to autism in terms of inflexible thinking and/or social misunderstanding of fluidity and difference between gender identity, gender role, and norms.**	3.00	7.00	5.60	0.94	Important	Yes	75
**Clinicians should ask the individual for their insights on the internal experience and meaning of their behaviors, which can help distinguish between features of autism and gender diversity.**	4.00	7.00	5.79	0.98	Important	Yes	74
**Longitudinal assessment may be needed to reach more diagnostic certainty in some gender-diverse individuals with more complex presentations.**	4.00	7.00	6.50	0.83	Extremely important	Yes	90
**Clinicians should be aware of subthreshold autism presentations being common in gender-diverse individuals, emphasizing a dimensional rather than categorical conceptualization of autism.**	5.00	7.00	6.30	0.80	Important	Yes	80
Clinicians should consider the individual’s gender transition status and how this relates to presentation in the autism assessment.	4.00	7.00	6.00	1.03	Important	No	80
Clinicians should assess for the impact of gender minority stress on social development, particularly in the presence of intellectual disability.	2.00	7.00	5.35	1.14	Slightly important	No	60
Rigidity in responses to misgendering can be indicative of autism.	2.00	6.00	4.55	1.36	Slightly important	No	35
Clinicians should be aware that many gender-diverse individuals have a strong social motivation that can mask difficulties in social communication, particularly in early development.	3.00	7.00	5.26	1.15	Slightly important	No	53
**It is important to use multiple sources for the developmental history (eg, caregivers, school).**	6.00	7.00	6.32	0.48	Important	Yes	100
**Clinicians need to reconceptualize and label the so-called female autism phenotype in their practice.**	6.00	7.00	6.15	0.37	Important	Yes	100
**Clinicians should be aware that high IQ may commonly intersect with autism and gender diversity, which can lead to later identification of autism due to compensation.**	5.00	7.00	5.95	0.51	Important	Yes	85

Across A criteria							
In people with social difficulties linked to gender diversity, clinicians should check for flexibility and variance based on social context or interaction partner. For example, does their social behavior change based on being in a situation in which they feel less safe to freely express their gender? Does their behavior change based on their interaction partner’s perspective on their gender identity and expression, or gender diversity more broadly?	1.00	7.00	5.79	1.18	Important	No	95
Clinicians should assess the level of awareness of how others perceive their gender expression and general interest in gender; low awareness could indicate core deficits in perspective taking and flexible nonverbal communication.	1.00	7.00	5.63	1.38	Important	No	68
Clinicians should assess whether social avoidance or detachment is due to observing gender behavior in others and/or hypervigilance due to gender minority stress and/or autism.	1.00	7.00	6.16	1.34	Important	No	95
Clinicians should assess whether an individual’s difficulties with social emotional insight are related to autism and/or impact of navigating high social demands of gender-related needs.	1.00	7.00	6.05	1.58	Important	No	84
Clinicians should assess whether differences in emotional expression and alexithymia are linked to (unmet) gender-related needs, gender-related medical treatments, and/or autism.	1.00	7.00	5.89	1.63	Important	No	79

A1							
Reduced sharing of experiences about gender, including emotions, could be due to autism or due to rejection of gender-related emotions by others.	1.00	6.00	4.85	1.14	Slightly important	No	25
Clinicians should be aware that transgender girls and women can be required to develop their social skills to adapt to their social context and therefore can superficially show social insight and elevated emotional expression. This is due to gendered expectations about social behavior in certain social contexts.	1.00	7.00	5.85	1.42	Important	No	75
**Clinicians should assess whether reduced sharing of one’s own experiences and interests is due to social anxiety linked to responses to one’s gender identity and expression and/or autism.**	4.00	7.00	5.95	0.60	Important	Yes	90
**Clinicians should assess whether less reciprocity in social interaction and communication is due to social anxiety, body dysphoria, and/or autism.**	4.00	7.00	6.45	0.94	Important	Yes	9
Clinicians should assess whether there is less enjoyment of social interactions due to gender minority stress and/or autism.	1.00	7.00	5.55	1.32	Important	No	75%

A2							
Clinicians should be aware that being gender diverse can influence nonverbal communication (eg, gesture, eye contact, tone of voice) due to studying and copying behavior of people of the same gender. This could lead to nonverbal communication that appears rehearsed, rigid, irregular, fluctuating, or exaggerated or could be improved due to effort.	1.00	7.00	5.75	1.25	Important	No	80
Clinicians should assess awareness, understanding, and anxiety about nonverbal cues of gender norms in nonverbal behavior (eg, posture, walking gait, gesture) and motivation and strategies to adhere to these to disentangle gender-related needs and autism.	1.00	6.00	5.10	1.17	Slightly important	No	40
**Clinicians should assess if unusual quality of speech (eg, tone, rate, modulation) is due to discomfort with voice and/or autism.**	5.00	7.00	6.00	0.46	Important	Yes	9
**Clinicians should assess whether limited eye contact is due to social anxiety, gender dysphoria, or gender minority stress associated with gender diversity and/or is due to autism.**	5.00	7.00	5.90	0.55	Important	Yes	80
**Clinicians should be aware of the effortful use of intonation in individuals wanting to be perceived by others as being their gender (to pass), which is often insufficiently coordinated with speech content in autistic individuals.**	4.00	7.00	5.80	0.77	Important	Yes	80
Clinicians should consider if less fluent nonverbal communication is due to social anxiety linked to gender minority stress and/or due to autism.	1.00	7.00	5.25	1.21	Slightly important	No	45
Clinicians should assess whether reduced use of gesture is due to social anxiety and gender minority stress and/or autism.	1.00	6.00	4.90	1.07	Slightly important	No	20

A3							
**Clinicians should assess whether social withdrawal and poor quality of social relationships is due to experiences of bullying, rejection, and dysphoria (eg, voice, body) and/or due to autism.**	5.00	7.00	6.20	0.52	Important	Yes	95
**Clinicians should assess quality over quantity of relationships—discomfort in one-to-one interactions, preference for online relationships, or reduced opportunity or reward can limit the number of friendships in gender-diverse individuals, even in the absence of autism.**	4.00	7.00	5.95	0.60	Important	Yes	90
Clinicians should assess whether atypical play was linked to a drive for and possible diminished opportunity to experience gender-related imaginative play and/or autism.	2.00	6.00	4.75	0.97	Slightly important	Yes	20
Clinicians should assess whether absence of interest in or isolation from peers could be driven by gender diversity, particularly if there was limited support for play with chosen playmates of the same gender in childhood, and/or these social differences are due to autism.	1.00	7.00	5.80	1.24	Important	No	85
**Clinicians should assess whether difficulties initiating and maintaining friendships is due to fear of rejection and discrimination and/or is part of a core difficulty related to autism in neurotypical contexts.**	5.00	7.00	6.55	0.60	Extremely important	Yes	95
**Clinicians should be aware that supportive spaces, for example, within the gender-diverse community, can facilitate better relationships. Therefore, clinicians need to assess current and development of social insight and the extent that friendships are mutual, symmetric, and maintained in the context of facilitation of supportive or nonsupportive environments.**	4.00	6.00	5.80	0.52	Important	Yes	85
Clinicians should assess whether the development of ability to understand and maintain relationships has been impaired by family or peer pressure to have only same-sex friendships in a gender-diverse individual.	1.00	7.00	5.35	1.27	Slightly important	No	60
**Clinicians should consider whether the individual’s ability to adjust behavior to social contexts is impaired by dysphoria (ie, low mood and anxiety) related to gender diversity and/or due to autism.**	4.00	7.00	5.50	0.83	Important	Yes	50

Across B criteria							
Clinicians should assess whether ritualized or atypical nonverbal behavior could be related to gender expression and appearance (eg, hair smoothing) and/or autism.	1.00	7.00	5.26	1.24	Slightly important	No	47
Clinicians should assess whether stereotyped behaviors such as stimming are suppressed due to gender-diverse individuals having an increased motivation to fit in.	1.00	7.00	4.84	1.26	Slightly important	No	21

B1							
Clinicians should assess whether repetitive or stereotyped language is related to gender expression and/or sameness behavior in the context of autism.	1.00	6.00	4.05	0.97	Neutral	Yes	5

B2							
Clinicians should be aware of ritualized quality of routines when associated with gender, which can be missed as a feature of autism when the clinician assumes that the routine is related to gender (but is in fact a part of autism).	4.00	7.00	5.26	0.81	Slightly important	Yes	26
Clinicians should assess whether behavioral inflexibility is due to the need for specific gender expression (eg, clothing) and/or autism.	1.00	7.00	5.05	1.31	Slightly important	No	42
**Clinicians should assess whether ritualized and repetitive behaviors are linked to anxiety due to gender minority stress and/or autism.**	4.00	7.00	5.58	0.69	Important	Yes	58
**Clinicians should assess whether avoidance of new situations and new people is due to gender minority distress and dysphoria and/or related to sameness needs of autism.**	4.00	7.00	5.58	0.84	Important	Yes	58

B3							
Clinicians should perform a careful assessment of focused interests, including their intensity and how unusual these are. This will help to separate autistic focused interests from focus on gender due to gender diversity.	1.00	7.00	5.68	1.38	Important	No	74

B4							
Clinicians should assess whether sensory hypersensitivity is linked to gender minority stress, body dysphoria, and/or autism.	2.00	7.00	5.32	1.16	Slightly important	No	58
**Clinicians should be aware that autistic traits can be amplified by the interaction between hyper- or hyposensory arousal and body dysphoria.**	4.00	7.00	6.26	0.87	Important	Yes	84
**Clinicians should assess whether choice of clothes is linked to gender expression and/or sensory sensitivities.**	4.00	7.00	5.95	0.71	Important	Yes	8
Clinicians should assess whether sensory hyposensitivity is linked to body dysphoria or autism.	1.00	7.00	5.53	1.22	Important	No	68

Criterion C							
**Clinicians should conduct a thorough early history taking of early social, emotional, communication, and gender development to differentiate respective and intersecting roles of gender and social communication differences, as well as their trajectories over time.**	6.00	7.00	6.58	0.51	Extremely important	Yes	100
**Clinicians should be aware that puberty onset and social demands typically increase at the same time, causing challenges separating increases in gender distress and social communication difficulties.**	5.00	7.00	6.63	0.60	Extremely important	Yes	95

Criterion D							
**As much as possible, the clinician should understand the different contributions of autism, gender diversity, and mental health needs to functioning.**	5.00	7.00	6.21	0.63	Important	Yes	89

Criterion E							
Clinicians need to consider the role of intellectual disability and how this can increase experiences such as rigid thinking and difficulties sharing emotional experiences in gender-diverse individuals.	3.00	7.00	5.37	1.07	Slightly important	No	42

Practical Considerations							
**During the assessment, the individual needs to feel safe and comfortable. This allows for rapport building to conduct a valid assessment of the features of autism, particularly social interaction and focused interests, as well as other important factors including masking and mental health needs. To do this, an early conversation is needed to clarify the individual’s name and pronouns while asserting that it is okay for these to change, alongside a plan for working with any informants who may use different names/pronouns for the individual.**	4.00	7.00	6.79	0.71	Extremely important	Yes	95
**To ensure the individual is comfortable, the physical clinic environment should be considered (eg, with gender-neutral toilets available and LGBTQ+ awareness demonstrated through flyers and posters in the waiting area).**	5.00	7.00	6.22	0.55	Important	Yes	94
**All team members (eg, including administrative and reception staff) should be trained in gender diversity awareness and should be accepting of gender-nonconforming behaviors.**	4.00	7.00	6.74	0.73	Extremely important	Yes	95
**Assessing clinicians should work in a multidisciplinary team and be competent and knowledgeable regarding the intersection of gender diversity, mental health, and autism. They should be particularly skilled in differential diagnosis.**	6.00	7.00	6.47	0.51	Important	Yes	100
**Clinicians need to interpret standardized measures, including sex-related norms, with caution as these measures are not worded or normed for gender-diverse individuals.**	6.00	7.00	6.21	0.42	Important	Yes	100
**Clinicians should provide thorough support after diagnosis and support for individuals not diagnosed with autism.**	4.00	7.00	6.16	0.76	Important	Yes	89
**Clinicians should pay attention to gender-related questions and/or support needs within the autism assessment report.**	5.00	7.00	6.58	0.61	Extremely important	Yes	95
**Clinicians should maintain awareness of the complexities of gaining informed consent for the autism assessment and the potential consequences of an autism diagnosis for gender-diverse individuals.**	6.00	7.00	6.16	0.37	Important	Yes	100
**The outcome of the assessment should be discussed in relation to gender-related questions with the individual and family.**	4.00	7.00	6.53	0.77	Extremely important	Yes	95%
**Clinicians should make clear the consideration of name and pronouns in the assessment and report when a youth’s gender identity is not settled or when family members have different views about the youth’s gender identity.**	6.00	7.00	6.37	0.50	Important	Yes	100

Note: ADHD = attention-deficit/hyperactivity disorder; LGBTQ+ = lesbian, gay, bisexual, transgender, queer or questioning, and others; Max = maximum; Min = minimum; OCD = obsessive-compulsive disorder; PTSD = posttraumatic stress disorder.

a Boldface statements were rated as important or extremely important, and consensus was reached.

b Ratings: Extremely unimportant = 1-1.49; unimportant = 1.5-2.49; slightly unimportant = 2.5-3.49; neutral = 3.5-4.49; slightly important = 4.5-5.49; important = 5.5-6.49; extremely important = 6.5-7.

General considerations aimed to capture issues linked to diagnosis that were relevant across different diagnostic criteria, for example, “Clinicians should ask the individual for their insights on the internal experience and meaning of their behaviors, which can help distinguish between features of autism and gender diversity.” The across A and B categories were for statements that could not be tied to a specific subdomain of the criteria but were specifically linked to either overall social communication (eg, “Clinicians should assess whether social avoidance or detachment is due to observing gender behavior in others and/or hypervigilance due to gender minority stress, and/or due to autism”) or overall RRBIs, eg, “Clinicians should assess whether ritualized or atypical nonverbal behavior could be related to gender expression and appearance, eg, hair smoothing, and/or autism.” The practical considerations focused on adaptations that might be needed to facilitate a high-quality assessment (eg, “Clinicians should pay attention to gender-related questions and/or support needs within the autism assessment report”).

Survey two was completed by 20 experts (95% completion rate), with a different participant missing in this round from survey one. See Table 2 for a summary of all the statements, their average rating, and whether consensus was reached; statements that were rated on average as “important” or “extremely important” (scored 6 or 7) and for which a consensus was reached (SD <1.0) are in boldface. A total of 37 statements (57%) met these criteria. Notably, many statements were given a wide range of importance ratings by experts, with multiple statements given ratings ranging between “extremely unimportant” to “extremely important,” demonstrating the range of views in this complex clinical area.

A brief description of statements rated as at least “important” and that reached our definition of consensus is presented here. General considerations regarding consensus can be summarized as follows. Experts highlighted the need for differential diagnosis, acknowledging that other neurodevelopmental conditions and mental health needs are prevalent in gender-diverse individuals. Clinicians should maintain awareness of their own and societal gender stereotypes; beliefs about the autism phenotype in individuals assigned female at birth; and how these stereotypes might influence expectations regarding social communication, relationships, RRBIs, and sensory sensitivities. Clinicians need to understand masking of autistic features and modifications to gender expression to reduce the likelihood of the person being misgendered. Rigid thinking about gender could be influenced by social differences or by gender-related needs. Understanding the person’s internal experience and meaning of behaviors, longitudinal assessments, and gathering the developmental history from multiple sources may help in distinguishing autistic features from developmental differences linked to gender diversity. Cognitive capabilities (eg, high IQ) leading to compensation behaviors and subthreshold autism presentations may be particularly prominent in this population.

Regarding the *DSM-5-TR* A criteria of social communication and interaction differences, experts highlighted the need to parse the potential impact of social anxiety and gender dysphoria in reduced sharing of experiences and interests and reduced reciprocity in social interaction. Differences in tone, rate, modulation, and intonation of speech and eye contact could be linked to gender expression and dysphoria and/or autism. The role of discrimination and rejection due to being gender diverse was highlighted as potentially contributing to social withdrawal and difficulties initiating and maintaining friendships, as well as the impact low mood and anxiety could have on a person’s ability to flexibly adjust their behavior according to the social context. Experts also highlighted the need to focus on quality over quantity of friendships due to potential difficulties in developing wide social networks experienced by gender-diverse individuals. Finally, a focus on variability of functioning across social contexts is crucial as some social spaces are more welcoming and accommodating to gender-diverse individuals than other spaces.

Regarding the *DSM-5-TR* B criteria for RRBIs, experts highlighted the need to understand if ritualized and repetitive behaviors and avoidance of new situations and people are driven by anxiety due to gender minority stress or autism-related needs. The role of body dysphoria in amplifying hyper- or hyposensory arousal was raised. Finally, choice of clothes could be due to sensory sensitivities and/or gender expression.

To assess whether autism features were present in early development, experts suggested a thorough early history taking to include gender identity development and related behaviors to understand the intersecting role of gender identity with autism-related developmental trajectories. Further, clinicians need to understand that puberty can increase gender dysphoria and is accompanied by increased social demands in adolescence, which can cause difficulties in separating gender distress and social difficulties. To understand functional impact, clinicians should aim to understand different contributions of autism, gender diversity, and mental health on functioning, insofar as this is possible.

Finally, there were many practical considerations. For a valid assessment, the gender-diverse person must feel safe and comfortable; early conversations are needed about the person’s name and pronouns, while asserting it is okay for these to change, alongside having a plan for informants who use different names/pronouns for the person. The clinic environment should be comfortable for gender-diverse people (eg, provide gender-neutral toilets), and staff should be trained in supporting this group (including administrative and reception staff). Clinical staff should be competent in differential diagnosis, including the intersections of autism, gender diversity, and mental health. Standardized measures should be interpreted with caution given the lack of norms for this population. Support should be offered to all clients after the assessment, including those not diagnosed with autism. Gender-related needs should be considered in the autism assessment report and feedback sessions, with clinicians understanding the impact of these assessments for accessing gender-related health care in their local context. Consideration should be given to the use of name and pronouns for individuals in situations in which their gender identity is not settled or, in the case of youth, in which parents/carers have different views about the youth’s gender identity.

## Discussion

### Summary of Findings and Links to the Wider Literature

This modified Delphi study resulted in 37 consensus statements that were rated on average by experts as “important” or “extremely important” for autism diagnostic assessments with gender-diverse individuals. These statements were categorized as being general considerations, linked to the *DSM-5-TR* criteria (A-E), or practical considerations for the gender-diverse population. Table 3 contains a lay summary of each statement to provide a clear explanation of each statement to guide clinical practice. These summaries were generated through collating and summarizing the qualitative comments from survey one of the Delphi procedure to allow an expanded, detailed explanation of the important ideas provided by the experts. It is important to note that there is currently a paucity of evidence to guide clinical practice in this area.^5^ These final recommendations are based on clinician expertise; they should be considered as providing preliminary clinical guidance while generating hypotheses to be tested in future empirical studies. These statements were created to accompany existing best practice autism diagnostic assessment guidelines.^28^, ^29^, ^30^, ^31^

**Table 3:** Considerations in Practice: Autism Diagnostic Assessments With Gender-Diverse Individuals

General considerations	Practice summary
Particular attention must be paid to disentangling autism from (co-)occurring developmental (eg, intellectual disability, ADHD) and mental health needs. This may include considering the roles of depression, anxiety (especially social anxiety), OCD, personality disorders, trauma responses, and PTSD and how these influence history, current presentation, and functioning.	Conducting a thorough differential diagnostic process is particularly important for this population. Clinicians should carefully assess for coexisting conditions. Gender-diverse individuals are at greater risks of mental health problems compared with cisgender people. Further, additional neurodevelopmental conditions such as ADHD, learning disabilities, or intellectual disabilities may be present. Clinicians must ensure that the identified differences in social communication and interaction, as well as RRBIs, are not better accounted for by the presence of other conditions.
Clinicians must maintain awareness of their own and societal gender stereotypes. They should also be aware of sex- and gender-related differences in social communication styles, social relationships, and restricted and repetitive behaviors and sensory sensitivities.	Clinicians may hold particular views about typical behaviors in boys and men, girls and women, and nonbinary individuals. Clinicians should strive to be aware of their own beliefs and cultural norms about how sex- and gender-related differences could influence an individual’s presentation at the assessment and the clinician’s interpretation of the presentation. For example, men might be expected to display fewer gestures, which could influence the presentation and/or clinician interpretation of their presentation during assessment of individuals with masculine identities. Limited gestures should not be interpreted as an autistic behavior if this is because an individual is modifying their behavior to appear more masculine or if the clinician has made a prediction based on gender stereotypes about a woman, whom they would expect to gesture more.
Clinicians should aim to understand the extent to which masking is autism and/or gender related (ie, modifying one’s gender expression to conform to social norms regarding typical gender behavior—to be seen as gender conforming). Because gender masking can impact social development, clinicians should explore the impact of any gender-related masking on sense of self and social development.	Individuals being assessed may modify their behaviors to fit in with others, which could cause difficulties in assessing autism-related behaviors and experiences. If an individual modifies their gender expression throughout development, this could limit their social development; for example, it could mean that they struggle to develop friendships or find social interactions tiring due to engaging in efforts to pass as their gender. Therefore, being gender diverse could contribute to differences in social development that are not due to being autistic.
Clinicians should assess if rigid thinking about gender could be linked to gender diversity and gender-related needs or is more related to autism in terms of inflexible thinking and/or social misunderstanding of fluidity and difference between gender identity, gender role, and norms.	Clinicians need to be cautious when interpreting rigid thinking as related to gender. Gender-diverse individuals may experience perfectionistic and inflexible thinking about gender expression linked to the stage of their gender journey. Conversely, rigid thinking about gender, particularly as it relates to understanding perspectives of other people on gender and social norms and thinking flexibly about gender, could be linked to autism.
Clinicians should ask the individual for their insights on the internal experience and meaning of their behaviors, which can help distinguish between features of autism and gender diversity.	It is important to listen to the individual’s own experiences and the meaning of their behaviors. This will help clinicians understand whether they meet criteria for autism. For example, if they explain that their repetitive behavior of smoothing their hair is linked to anxiety about their hair appearing feminine enough, this should not be recorded as an autistic behavior.
Longitudinal assessment may be needed to reach more diagnostic certainty in some gender-diverse individuals with more complex presentations.	Clinicians may need to take more time to develop a thorough understanding of the individual. Assessing social communication behaviors and RRBIs longitudinally may be necessary to understand how possible features of autism interact with gender identity and its development over time. For example, it may be necessary to assess someone again when they are experiencing less gender dysphoria, as significant distress about gender could reduce an individual’s willingness and capability to interact with others, leading to an inaccurate diagnosis of autism.
Clinicians should be aware of subthreshold autism presentations being common in gender-diverse individuals, emphasizing a dimensional rather than categorical conceptualization of autism.	Individuals may have high autism traits but not reach the threshold for an autism diagnosis based on *DSM-5-TR*. Therefore, emphasizing the idea of an autism spectrum might be helpful for individuals with high autism traits who do not meet the full diagnostic criteria.
It is important to use multiple sources for the developmental history (eg, caregivers, school).	Given the complexity of these assessments, it is important to triangulate information from multiple sources. In the case of youth, this may be from teachers, parents/caregivers, and other significant adults.
Clinicians need to reconceptualize and label the so-called female autism phenotype in their practice.	The terminology regarding the female autism phenotype can be offensive and exclude gender-diverse individuals, so clinicians should be wary about using this terminology. This conceptualization is also stereotypical and can be problematic in implying an exclusive link between specific presentations and a specific sex or gender. It is possible for an autistic individual to display this nuanced pattern of autistic behaviors and experiences but not identify as female. More appropriate terminology may be nuanced autism phenotype.^22^
Clinicians should be aware that high IQ may commonly intersect with autism and gender diversity, which can lead to later identification of autism due to compensation.	Gender-diverse individuals undergoing autism assessments are particularly likely to be highly intelligent. This may contribute to later identification of autism due to high rates of compensation.

A1	
Clinicians should assess whether reduced sharing of one’s own experiences and interests is due to social anxiety linked to responses to one’s gender identity and expression and/or autism.	Gender-diverse individuals may not feel safe when sharing their experiences and interests, so clinicians should be cautious when interpreting a lack of reciprocity in this group.
Clinicians should assess whether less reciprocity in social interaction and communication is due to social anxiety, body dysphoria, and/or autism.	Gender-diverse individuals are more likely to experience social anxiety and distress about their bodies, both of which are associated with increased risk of depression, and these may contribute to limited social reciprocity due to mental health rather than autism.

A2	
Clinicians should assess if unusual quality of speech (eg, tone, rate, modulation) is due to discomfort with voice and/or autism.	Clinicians should ensure they understand the reasons for differences in quality of speech in individuals they assess. Gender-diverse individuals may have heightened awareness of gendered interpretation of the way they talk. This could lead to modifying their voice and way of speaking so that others accurately identify their gender: this could include the pitch, emphasis, and intonation of voice, as well as speed of talking.
Clinicians should assess whether limited eye contact is due to social anxiety, gender dysphoria, or gender minority stress associated with gender diversity and/or is due to autism.	There are multiple reasons why a person might engage in limited eye contact that are not due to being autistic. Individuals with social anxiety may avoid eye contact in an attempt to reduce their experience of anxiety. Individuals experiencing gender dysphoria may reduce eye contact due to high levels of distress or concerns about how others will perceive their gender. Gender-diverse individuals can experience stigma and discrimination due to their gender identities (ie, gender minority stress), and this could affect their willingness and capability to use eye contact in social interactions due to previous adverse experiences.
Clinicians should be aware of the effortful use of intonation in individuals wanting to be perceived by others as being their gender (to pass), which is often insufficiently coordinated with speech content in autistic individuals.	Gender-diverse individuals may effortfully modulate their speech intonation to be perceived by others as their gender due to gender stereotypes about how particular genders use speech intonation (eg, women may be expected to be more expressive and use a more singsong voice). If the individual is autistic, their modulation may be mismatched with the content of their speech.

A3	
Clinicians should assess whether social withdrawal and poor quality of social relationships is due to experiences of bullying, rejection, and dysphoria (eg, voice, body) and/or due to autism.	Clinicians should interpret difficulties with social relationships with caution. Gender-diverse individuals may reduce their social contact and experience reduced quality of social relationships due to adverse social experiences and current distress.
Clinicians should assess quality over quantity of relationships—discomfort in one-to-one interactions, preference for online relationships, or reduced opportunity or reward can limit the number of friendships in gender-diverse individuals, even in the absence of autism.	To understand whether an individual’s difficulties in relationships are driven by gender minority stress or autism, it is helpful to especially consider the quality of social relationships beyond the quantity. Gender-diverse individuals may have a lower number of higher-quality relationships. This could be due to preferring to socialize online, where it can be easier to be perceived as one’s gender identity; feeling unsafe in some social environments, limiting opportunities; or experiencing less rewarding social relationships due to the complexities of navigating friendships as a gender-diverse person.
Clinicians should assess whether difficulties initiating and maintaining friendships is due to fear of rejection and discrimination and/or is part of a core difficulty related to autism in neurotypical contexts.	Gender-diverse individuals may have difficulties with initiating or maintaining friendships due to experiences of gender minority stress and fears of not being accepted. This could be linked to previous negative experiences or fear of future negative experiences associated with being gender diverse rather than due to being autistic.
Clinicians should be aware that supportive spaces, for example, within the gender-diverse community, can facilitate better relationships. Therefore, clinicians need to assess current and development of social insight and the extent that friendships are mutual, symmetric, and maintained in the context of facilitation of supportive or nonsupportive environments.	Some environments can facilitate better social relationships for autistic people, and the gender-diverse community can provide one such environment. Clinicians need to be aware of this and assess the individual’s social insight and how reciprocal their friendships are. Are the friendships maintained out of the supportive environment? Does the individual still struggle with social understanding and interaction challenges despite the supportive environment? Understanding and contrasting these scenarios could indicate the extent to which the individual is struggling with social insight and relationships due to being autistic.
Clinicians should consider whether the individual’s ability to adjust behavior to social contexts is impaired by dysphoria (ie, low mood and anxiety) related to gender diversity and/or due to autism.	Low mood and anxiety related to gender dysphoria, rather than autism, might reduce the individual’s ability to flexibly respond to different social contexts.

B2	
Clinicians should assess whether ritualized and repetitive behaviors are linked to anxiety due to gender minority stress and/or autism.	Behaviors may appear ritualized when they are in fact an individual’s effortful attempt to be perceived by others as their gender identity. This could include ritualized nonverbal behaviors (eg, stereotypical masculine behaviors) or repetitive tidying of one’s appearance (eg, hair smoothing, adjusting clothes).
Clinicians should assess whether avoidance of new situations and new people is due to gender minority distress and dysphoria and/or related to sameness needs of autism.	Individuals may avoid new situations due to a fear of being misgendered in new contexts or due to insistence on sameness.

B4	
Clinicians should be aware that autistic traits can be amplified by the interaction between hyper- or hyposensory arousal and body dysphoria.	Gender-diverse people who are experiencing body dysphoria, ie, distress about body parts that are not aligned with their gender identity, may be particularly focused on or avoidant of these body parts. This can interact with sensory arousal linked to autism.
Clinicians should assess whether choice of clothes is linked to gender expression and/or sensory sensitivities.	Clinicians should understand whether clothing choice relates to gender, autism, or both. Gender-diverse individuals may choose particular clothes to express their gender identity or to hide particular body parts.

Criterion C	
Clinicians should conduct a thorough early history taking of early social, emotional, communication, and gender development to differentiate respective and intersecting roles of gender and social communication differences, as well as their trajectories over time.	To understand the relationships between social, emotional, communication, and gender development, clinicians could conduct a timeline, with autism-related development above the line and gender-related development below the line to understand how these experiences relate to one another over time.
Clinicians should be aware that puberty onset and social demands typically increase at the same time, causing challenges separating increases in gender distress and social communication difficulties.	As puberty can present particular challenges to both autistic and gender-diverse individuals, clinicians should pay careful attention to the individual’s experiences at this point in their developmental history. Puberty can be challenging for autistic people due to the unpredictable body changes and associated sensory experiences. Further, the social landscape changes at this age, and social demands increase, including expectations and motivations regarding romantic relationships and increases in gender stereotypical behavior in most adolescents. Puberty can be challenging for gender-diverse individuals due to their body changing in an unwanted way and emerging sexuality. Clinicians need to understand how the youth made sense of any difficulties experienced at this time as well as their impact.

Criterion D	
As much as possible, the clinician should understand the different contributions of autism, gender diversity, and mental health needs to functioning.	It can be particularly challenging to delineate the impact of different intersecting identities and experiences as well as experienced neurodevelopmental or mental health challenges on an individual’s functioning. To ensure that the individual truly meets the criteria for autism, clinicians should try to understand how different experiences impact functioning. For example, is attending college difficult due to social anxiety, fear of being misgendered, avoidance of new environments, social and/or sensory overload, or a combination of these?

Practical considerations	
During the assessment, the individual needs to feel safe and comfortable. This allows for rapport building to conduct a valid assessment of the features of autism, particularly social interaction and focused interests, as well as other important factors including masking and mental health needs. To do this, an early conversation is needed to clarify the individual’s name and pronouns while asserting that it is okay for these to change, alongside a plan for working with any informants who may use different names/pronouns for the individual.	Gender-diverse individuals often have experiences of being made to feel uncomfortable and/or unsafe. Clinicians must be aware of this and do everything possible to ensure that the individual feels safe and comfortable. This means using the individual’s chosen name and pronouns and developing a shared plan for working with informants who may use a different name/pronouns. Only then can a valid assessment be conducted.
To ensure the individual is comfortable, the physical clinic environment should be considered (eg, with gender-neutral toilets available and LGBTQ+ awareness demonstrated through flyers and posters in the waiting area).	The individual is likely to be more comfortable if simple adjustments are made to the environment, including demonstrating an awareness of LGBTQ+ issues, offering welcoming messages, and providing gender-neutral toilets.
All team members (eg, including administrative and reception staff) should be trained in gender diversity awareness and should be accepting of gender-nonconforming behaviors.	Gender diversity awareness training for all team members will ensure the comfort of the individual being assessed.
Assessing clinicians should work in a multidisciplinary team and be competent and knowledgeable regarding the intersection of gender diversity, mental health, and autism. They should be particularly skilled in differential diagnosis.	Assessments with gender-diverse individuals can be particularly complex. To ensure a high-quality assessment can be conducted, team members should be from multiple professional backgrounds, and there must be expertise in autism, gender diversity, and mental health, with strong differential diagnosis skills.
Clinicians need to interpret standardized measures, including sex-related norms, with caution as these measures are not worded or normed for gender-diverse individuals.	Standardized measures and assessment tools for autism have not been developed specifically for gender-diverse individuals. This means that scores should be interpreted with caution and in the context of the whole assessment and all information collected before a conclusion is reached.
Clinicians should provide thorough support after diagnosis and support for individuals not diagnosed with autism.	All individuals assessed for autism are likely to have additional needs that should be supported following the assessment. Individuals who do not meet criteria for autism are still likely to need support. Therefore, support after the assessment should be offered to all individuals to maximize positive outcomes following the demanding and resource-intensive assessment process.
Clinicians should pay attention to gender-related questions and/or support needs within the autism assessment report.	When writing the autism assessment report, clinicians should acknowledge gender-related questions (eg, that identity or gender-related preferences may change in the future, considering autism-related needs within gender-related health care, information processing profile and how this might affect communication about gender-related health care).
Clinicians should maintain awareness of the complexities of gaining informed consent for the autism assessment and the potential consequences of an autism diagnosis for gender-diverse individuals.	Having an autism diagnosis can impact access to gender-related health care in some settings. Therefore, clinicians must be aware of their particular setting and how this could influence access to gender-related health care. For example, in some jurisdictions, it might not be possible to access gender-related health care until the autism assessment is complete. In other jurisdictions, receiving an autism diagnosis could limit access to gender-related health care. These factors could influence an individual’s motivation for assessment and presentation during the process.
The outcome of the assessment should be discussed in relation to gender-related questions with the individual and family.	The clinician should give the individual (and their family when appropriate) the opportunity to discuss the outcome of the autism assessment and how this could relate to gender-related questions.
Clinicians should make clear the consideration of name and pronouns in the assessment and report when a youth’s gender identity is not settled or when family members have different views about the youth’s gender identity.	Clinicians must be sensitive to situations in which the individual’s gender identity is not settled or when different parts of the support network (such as the family) have different views to write a respectful and accurate report. Ideally, an open dialogue with all people contributing will facilitate a respectful and person-centered dialogue, report, and recommendation.

Note: ADHD = attention-deficit/hyperactivity disorder; LGBTQ+ = lesbian, gay, bisexual, transgender, queer or questioning, and others; OCD = obsessive-compulsive disorder; PTSD = posttraumatic stress disorder; RRBI = restricted, repetitive behaviors, interests, and activities.

General considerations highlighted clinical complexity, with clinicians requiring a high level of knowledge of co-occurring conditions and nuanced autism presentations. Experts suggested aspects of the process leading to a more robust assessment, including gathering developmental history from multiple sources and longitudinal assessments. Practical considerations for obtaining a valid assessment included ensuring that the gender-diverse person feels comfortable throughout the process, that staff are qualified to conduct assessments, and that feedback is provided in a way that considers gender-related needs. This resonates with research indicating that both autistic^32^ and gender-diverse^33^ individuals can feel alienated and unsupported in mainstream health care settings, a finding corroborated by individuals who are both autistic and gender diverse.^34^

Experts generated more statements linked to social communication and interaction differences compared with RRBIs. Statements focused on the former included understanding the role of gender minority stress in social difficulties, as socializing can be challenging for gender-diverse individuals due to stigma, rather than because of innate social communication differences.^35^ Moreover, impression management efforts can contribute to differences in speech and eye contact that are not driven by the features of autism.^36^ There are likely nuances in such impression management efforts of which clinicians should be aware. First, individuals may mask to appear to be cisgender, which may be more about trying to fit in and avoid discrimination,^37^ just as individuals may attempt to hide that they are autistic to fit in.^36^^,^^38^ Second, individuals may modify their behaviors to be perceived as belonging to their gender group, to pass as their gender identity and be affirmed by others.^37^ In practice, this could mean a person studying behaviors of cisgender people or of people of the same gender identity and working hard to emulate such behaviors (eg, eye contact, gesture, voice pitch and modulation) with effortful monitoring of these behaviors, which could lead to differences in social communication behaviors that are not linked to autism. These different strategies may have different impacts on mental health^39^ as well as social communication differences seen in assessments. This resonates with qualitative work with autistic adults experiencing gender dysphoria in which participants described feeling different and experiencing discrimination and conflict regarding expressing their feelings about their gender identity.^40^

In terms of RRBIs, experts highlighted the need to understand if ritualized and repetitive behaviors and avoidance of new situations and people are driven by anxiety due to gender minority stress or autism-related needs, or both. The role of body dysphoria in amplifying sensory hyper- or hypoarousal was highlighted, aligning with qualitative work indicating these experiences can intersect and amplify one another in autistic and transgender youth and adults^41^ and quantitative work indicating higher rates of sensory sensitivities in gender-diverse individuals.^42^ Therefore, assessing how sensory processing differences have related to gender dysphoria, for example, whether and how sensory sensitivities focused on parts of the body are experienced as distressing, and understanding longitudinal trajectories of such distress could help clinicians elucidate whether such differences are related to autism, gender diversity, or both.

To assess whether features of autism were present in the early developmental period, experts emphasized that a thorough developmental history taking should include gender identity development and related behaviors to understand the intersecting role of gender identity with the emergence and change of possible autism features, including during puberty and adolescence. Puberty is a particularly challenging time for gender-diverse youth^43^ and autistic youth,^44^^,^^45^ and especially for those who are both gender diverse and autistic.^41^ This is also the developmental period when the increased clinical needs finally lead to a late autism assessment and diagnosis, if autism has not been recognized during childhood. As a clinical diagnosis must be supported by its functional impact, clinicians should clarify the differential contributions of potential autism, gender diversity, and mental health on functioning to aid diagnostic decision making.

Longitudinal research that analyzes the developmental associations between features of autism and gender diversity could significantly improve diagnostic reliability. Surprisingly, a comprehensive understanding of the factors contributing to contemporary gender identity development in general (eg, in nonautistic individuals) is lacking.^46^, ^47^, ^48^ To properly understand the autism–gender diversity intersection, we need strong longitudinal evidence about gender identity development in both autistic and nonautistic individuals.

Notably, there was more consensus about the generic considerations and adaptations during the assessment processes compared with the specific ways in which gender diversity could influence the development of particular social communication and RRBI differences. This is in line with the current limited knowledge about the intersection of autism and gender diversity,^49^ and it is therefore logical that clinicians are more aligned in their general approaches than in how specific aspects of the *DSM-5-TR* criteria for autism should be modulated for gender diversity.

The expert statements could be treated as promising hypotheses borne of clinical expertise globally that warrant empirical investigations. According to the experts we surveyed, 2 aspects of the experiences of gender-diverse individuals are particularly pertinent to an autism assessment. First, the experiences of gender minority stress^50^^,^^51^ may well impact a person’s social experiences, for example, if a child’s social opportunities are limited due to being ostracized for nonconforming gender-related presentations^10^ or because their preferred playmates are of the opposite sex. This could partially contribute to higher reported autistic traits and/or autism diagnoses in gender-diverse individuals. Furthermore, adverse social experiences increase the likelihood of social anxiety,^52^ which may lead to an atypical social communication style driven by safety-seeking behaviors,^53^ rather than by neurodevelopmental difference. Second, gender-diverse individuals wishing to pass as their gender identity may consciously modify certain aspects of their social communication, for example, voice pitch and modulation. This could contribute to idiosyncrasies in social communication presentation, which could be falsely attributed by diagnosticians to an autistic social communication profile. All these conceptualizations and plausible differentiation warrant further empirical validation, ideally comparing autistic and nonautistic individuals throughout the lifespan.

A key strength of this Delphi study was the participation of world experts in the field of autism and gender diversity, which has led to a list of preliminary consensual considerations for clinicians conducting autism assessments with gender-diverse people across ages. These statements could offer some initial guidance and prompt future empirical investigation and validation efforts.

However, there are important limitations, including that these considerations were generated only by clinicians, with limited involvement of gender-diverse and autistic people (who are not also diagnosticians) due to the technical and clinical nature of our research questions, which require in-depth knowledge of the intricacies of the autism diagnostic process. Future research including more gender-diverse and autistic clinicians, researchers, and nonclinician/researcher community members is needed. Our inclusion criteria allowed some individuals who had not conducted a large number of autism assessments with gender-diverse individuals to participate, so some participants may not have been as expert as intended. In addition, our sample was largely based in Europe (67%), limiting the generalizability of our findings. Further, the Delphi process aims to reach clinical consensus; however, we conducted a modified Delphi procedure and used existing definitions of consensus within the Delphi method.^27^ Whereas 37 statements met this a priori consensus definition, many statements were given vastly different ratings by different experts, indicating the diversity of opinions and approaches currently in this field and across different regions of the globe. As definitions of gender partly vary by context, it is perhaps inevitable that clinicians working in different sociocultural contexts have differing opinions on the intersection of gender diversity and autism. It is also entirely possible that other factors such as professional background (ie, those who are primarily clinicians vs primarily researchers, those who primarily work with autistic individuals vs those who primarily work with gender-diverse individuals), level of experience, clinician gender identity and neurodivergence status, and the sociocultural norms of the region where the clinicians are practicing affected the results, but it is beyond the capability of our current dataset, analyses, and qualitative study framework to provide informed and robust analyses to explore these factors. It is plausible that there are meaningful differences in this intersection cross-culturally that await further clarification.

Autism diagnostic assessments are complex clinical tasks, which can be particularly challenging to conduct for individuals with unique developmental experiences and conditions where robust, longitudinal evidence about how they developmentally intersect with the features of autism is lacking. The statements should be considered preliminary considerations for clinicians working in this field as they are based on expert opinions rather than more rigorous evidence-based medical research. Experts contributing to this Delphi study were most aligned on general and practical considerations (eg, using a longitudinal assessment, using multiple sources for developmental history, ensuring the person felt safe and comfortable during the assessment), whereas there was less cohesion in the specific intersection of *DSM-5-TR* criteria and gender diversity, which aligns with the current level of knowledge we have about this intersection.

## CRediT authorship contribution statement

**Kate Cooper:** Writing – review & editing, Writing – original draft, Methodology, Formal analysis, Data curation, Conceptualization. **Anna I.R. van der Miesen:** Writing – review & editing, Methodology, Formal analysis, Data curation, Conceptualization. **Meng-Chuan Lai:** Writing – review & editing, Methodology, Formal analysis, Data curation, Conceptualization.
